# A 22-Year-Old Female with Invasive Epithelioid Angiomyolipoma and Tumor Thrombus into the Inferior Vena Cava: Case Report and Literature Review

**DOI:** 10.1155/2013/730369

**Published:** 2013-07-25

**Authors:** Campbell Grant, John M. Lacy, Stephen E. Strup

**Affiliations:** ^1^University of Kentucky College of Medicine, Lexington, KY 40536, USA; ^2^Division of Urology, Department of Surgery, University of Kentucky College of Medicine, Lexington, KY 40536, USA

## Abstract

A 22-year-old female presented with back pain and was discovered to have a right-sided abdominal mass. Computed tomography (CT) scan revealed a 9 cm enhancing right upper pole renal mass with suspicion for tumor thrombus into the right renal vein and possibly the inferior vena cava (IVC). Magnetic resonance imaging (MRI) confirmed tumor thrombus into the inferior vena cava approximately 3 cm below the hepatic venous confluence. Open right radical nephrectomy with inferior vena cava thrombectomy was performed with removal of right kidney and tumor thrombus en bloc. Pathology revealed malignant epithelioid angiomyolipoma (EAML or PEComa). Epithelioid angiomyolipoma is a rare tumor of mesenchymal tissue that has the potential for local invasion and disease progression. Diagnosis of EAML was confirmed by pathology and immunohistochemistry. She was referred to medical oncology for discussion of surveillance versus potential adjuvant therapy and ultimately opted for close surveillance.

## 1. Introduction

EAML is a potentially malignant mesenchymal mass closely related to angiomyolipoma and is composed of perivascular epithelioid, vascular, adipose, and smooth muscle cells. EAML may be or is associated with tuberous sclerosis in about 25% of cases. So far, only a small number of EAMLs have been reported. Here we report the case of a 22-year-old woman with a right-sided renal tumor and IVC tumor thrombus that was confirmed as an EAML with intermediate risk for progression by pathology.

## 2. Case Presentation

**Figure 1:**
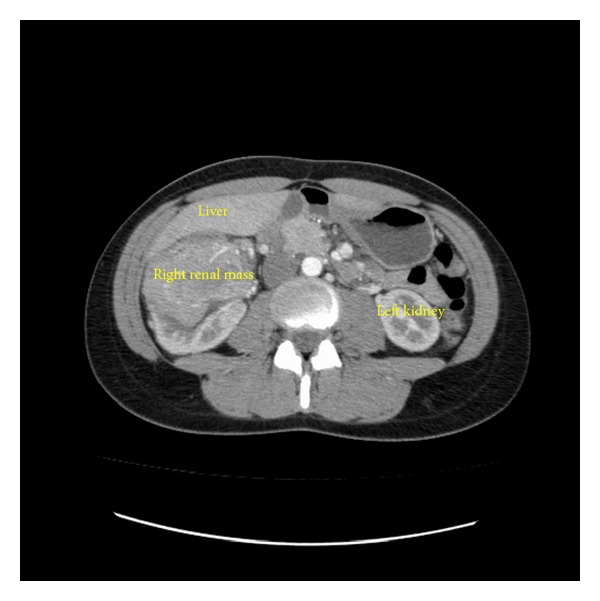
Axial images of computed tomography scan showing 9 cm right upper pole mass.

**Figure 2:**
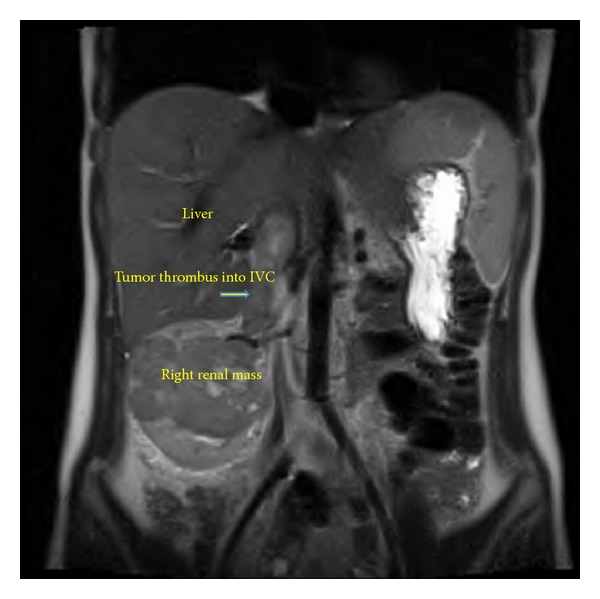
Coronal images of magnetic resonance imaging scan showing tumor thrombus extending into inferior vena cava.

A 22-year-old female presented with back pain and her primary care physician palpated a right-sided abdominal mass. Family history was positive for renal cell carcinoma in her grandmother diagnosed at the age of 63 years old but was negative for tuberous sclerosis. Computed tomography (CT) scan of the chest/abdomen/pelvis revealed a 8-9 cm enhancing right upper pole renal mass with suspicion for tumor thrombus into the renal vein and IVC (Figure 1). There was no lymphadenopathy or pulmonary involvement. Magnetic resonance imaging (MRI) confirmed a Zincke Level II venous tumor thrombus into the inferior vena cava to approximately 3 cm below the hepatic venous confluence (Figure 2).

**Figure 3:**
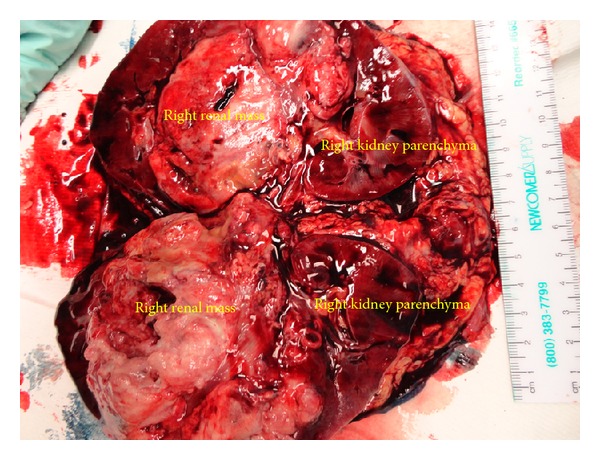
Gross specimen after en bloc removal and then transection by pathologist.

Open right radical nephrectomy with IVC thrombectomy was performed with removal of right kidney with mass and tumor thrombus en bloc (Figure 3). Transplant surgery was present and assisted with mobilization of the liver. 

Her postoperative course was complicated by bilateral pulmonary emboli. On postoperative day number two the patient had tachycardia and tachypnea. CT PE protocol revealed diffuse pulmonary emboli bilaterally. Systemic anticoagulation with a heparin drip was initiated and localized infusion of tissue plasminogen activator (tPA) was performed. The patient remained in the hospital until stable from a cardiac and respiratory standpoint and was discharged home on oral anticoagulation with warfarin.

Pathology revealed malignant epithelioid angiomyolipoma (PEComa), stage pT3bNx, Fuhrman grade IV. There was 1–50% tumor necrosis and extensive lymphovascular invasion, with involvement of the right renal vein and inferior vena cava as noted above, as well as renal sinus and perinephric fat invasion. Surgical margins were negative. The patient was referred to medical oncology to discuss close surveillance versus potential adjuvant chemotherapy. Based on her pathology, she is at intermediate risk for disease progression and ultimately chosen surveillance.

## 3. Discussion

Epithelioid angiomyolipomas (EAMLs) are a rare variant of angiomyolipomas with the capability of recurrence and metastasis. First reports of angiomyolipomas with epithelioid morphology were in 1995 and 1996 [1, 2]. Whereas renal angiomyolipomas are typically benign lesions, epithelioid angiomyolipomas are potentially malignant. Renal AML may sometimes show extension into the renal vein or IVC, but this is thought to be multifocal growth rather than metastasis [3]. Progressive enlargement and venous thrombosis are rare complications [4] and involvement of regional lymph nodes is uncommon [5]. AML is believed to belong to a family of lesions, characterized by proliferation of perivascular epithelioid cells. PEComas (tumors showing perivascular epithelioid cell differentiation) are a family of related mesenchymal neoplasms that include AML, LAM, and clear cell “sugar” tumor of the lung. There is a strong association between PEComas and tuberous sclerosis, which is due to the loss of tumor suppressor genes TSC1 or TSC2. Mutations of p53 tumor suppressor gene may also play some role in malignant transformation of EAMLs [6].

EAML is often confused with RCC on imaging. A typical renal AML is suggested by renal masses that are markedly hypoechoic relative to the renal parenchyma. Demonstration of fat within the tumor on CT imaging is thought to confirm the diagnosis [7]. Microscopically the presence of epithelioid cells can make differentiating from RCC difficult without immunohistochemistry.

EAML can only be definitively distinguished from benign AML by immunohistochemistry. EAML will be negative for cytokeratin and/or epithelial membrane antigen markers, and positive for one or more melanocytic markers and one or more actin markers [8, 9]. A recent study by Aydin arbitrarily qualified epithelioid tumors as containing at least 10% epithelioid cells [9]. One recent study demonstrated that estrogen receptor, PR, and bcl-2 markers were centered around dysmorphic vessels and were expressed more significantly in epithelioid AML variants [10].

Microscopically EAMLs are characterized by polygonal cells with clear to eosinophilic cytoplasm and round to oval nuclei that may show varying degree of nuclear atypia [9]. Pure epithelioid PEComas have two major architectural patterns: carcinoma like growth characterized by large cells arranged in cohesive nests, broad alveoli, and compartmentalized sheets separated by thin vascular-rich septae or epithelioid and plump spindle cells in diffuse growth arranged in diffuse sheets [8].

EAML is a potentially malignant variant of AML. A previous meta-analysis of 69 well-documented cases of EAML showed that 38% were malignant [11]. Attempts have been made to develop a risk stratification to determine progression of malignancy of EAMLs based on five parameters: (1) associated tuberous sclerosis complex, (2) necrosis, (3) tumor size greater than 7 cm, (4) extrarenal extension and/or renal vein involvement, and (5) carcinoma-like growth pattern. Of these 5 parameters, carcinoma-like growth pattern and extrarenal extension were the only reliable predictors of outcome [8]. The same study found that of the 33 cases of EAML studied, 10 of these had renal vein involvement, similar to our patient. Another study of 40 cases of EAML with atypia demonstrated several adverse histologic features associated with malignancy: large epithelioid cell component, severe nuclear atypia, extent of nuclear atypia, mitotic count, presence of atypical mitotic figures, necrosis, and the presence of lymphovascular invasion [12].

To date there has been little documented about how to treat the malignant version of EAML beyond excision. Chemotherapy has shown mixed results. There is one case report of doxorubicin reducing tumor size, but two other case reports showing poor outcome with chemotherapy regimens [13]. Kenerson et al. demonstrated that mTOR activation was common to non-TSC-related AMLs and PEComas and suggested that mTOR inhibitors such as rapamycin might be a potential avenue for treatment [14]. Due to the rare incidence of EAML, clinical trials to test this theory are unlikely; however, there are several case reports showing that rapamycin or other similar drugs may be clinically effective in reducing tumor size [15, 16].

In summary, our patient presented with a right renal mass with tumor thrombus into the renal vein and inferior vena cava, with final pathology revealing epithelioid angiomyolipoma. While rare, there have been several case reports on this type of tumor. Due to the rarity of this disease, there is currently a paucity of data regarding the need for adjuvant therapy or the potential responsiveness of these tumors to chemotherapy. Based on the current available data, this patient will remain on a close surveillance protocol with an intermediate risk of progression of disease. If she were to progress, mTOR inhibitors are one potential therapeutic option.
